# Comparison of the Effects of Endurance Training Conducted in Conditions of Normoxia and Artificial Hypoxia in Patients After Myocardial Infarction

**DOI:** 10.3390/jcm14061790

**Published:** 2025-03-07

**Authors:** Agata Nowak-Lis, Zbigniew Nowak, Dominika Grzybowska-Ganszczyk, Paweł Jastrzębski, Anna Konarska-Rawluk

**Affiliations:** 1 Department of Physiotherapy, Jerzy Kukuczka Academy of Physical Education, ul. Mikołowska 72a, 40-065 Katowice, Poland; z.nowak@awf.katowice.pl (Z.N.); dominikagrzybowska@yahoo.com (D.G.-G.); anko@int.pl (A.K.-R.); 2 Department of Cardiology, Leszek Giec Upper-Silesian Medical Centre, Medical University of Silesia, ul. Bielska 4, 43-400 Cieszyn, Poland; paweljastrzebski@mp.pl

**Keywords:** hypoxia, normobaric hypoxia, myocardial infarction, training, rehabilitation

## Abstract

**Background/Objective**: Attention should be paid to the introduction of more functional training methods during the second stage of cardiac rehabilitation, which imitate everyday activities to some extent. The main purpose of this research was to analyze the effects of a 22-day training program carried out in normobaric hypoxic conditions corresponding to the altitude of 3000 m a.s.l. in patients after myocardial infarction and to compare it with the same training conducted in normoxic conditions. **Material and Methods**: This study included 36 patients after myocardial infarction who underwent percutaneous angioplasty with stent implantation. They were examined before and after 2 days of training sessions: day one, spiroergometric exercise test on a mechanical treadmill, blood collection for laboratory tests; day two, echocardiography of the heart. Than patients underwent 22 days of training in hypoxic conditions. At the end of experiment patients had the same examinations as day one and two. **Results**: Training conducted in hypoxic conditions had a wider impact on spiroergometrical parameters. Significant, beneficial changes were demonstrated in relation to test duration, distance covered, energy expenditure MET, respiratory exchange ratio RER, as well as resting values of systolic and diastolic blood pressure. There were no changes in parameters for morphology, cytokines, and fibrinogen. There were some differences in relation to echocardiography examinations. **Conclusions**: The conditions in which the rehabilitation training was conducted affect the level of exercise tolerance. The hypoxic conditions in which the training was conducted affected only two hemodynamic parameters: LVESd and e’ septal. Rehabilitation training conducted in various environmental conditions had an impact only on the IL-10 value.

## 1. Background

The huge progress in recent years in the field of cardiology, both in diagnostics and treatment methods, has meant that patients leaving the hospital are often in excellent physical shape. This enables them to take on a number of challenges in the field of physical activity. Therefore, attention should be paid to the introduction of more functional training methods during the second stage of cardiac rehabilitation, which imitate everyday activities to some extent. One such potential training method is endurance training performed in conditions of artificial hypoxia (normobaric). Not only do hypoxic conditions provide the opportunity for more effective training, but these conditions can also provide an answer to a frequent question asked by patients: Is it safe to go to the mountains after a heart attack? Recently, several interesting scientific reports have been published, presenting the results of research conducted on a group of patients with coronary artery disease exposed to high altitudes [1,2]. The results showed that staying in hypoxic conditions causes the dilation of coronary and peripheral vessels, reduces the development of atherosclerosis, and reduces vessel stiffness, positively affecting blood pressure levels. According to Schmid et al., short-term exposure to an altitude of 3454 m above sea level (a.s.l.) is safe for patients with stable coronary artery disease and normal left ventricular function within 6 months after acute coronary syndrome or coronary artery bypass surgery [3]. The condition is a negative (without signs of ischemia) stress test result. Physical effort performed in hypoxic conditions may therefore prove to be extremely helpful during cardiac rehabilitation. However, choosing the right height above sea level is important for safety.

## 2. Objective

The progress in the treatment of patients after myocardial infarction has led to a situation where patients discharged from the hospital are often in excellent condition. This enables them to undertake various physical activity challenges. In many cardiac rehabilitation centers, methods that are relatively conservative compared to the patients’ capabilities are used. These approaches often lead to discouragement from further exercise, as they tend to be monotonous and less engaging. Therefore, attention should be given to implementing more functional forms of cardiac rehabilitation that can, to some extent, mimic daily life activities. One such method is endurance training under normobaric hypoxic conditions. This approach not only allows for more effective training but may provide an answer to a question frequently asked by patients: Is it safe to travel to the mountains after experiencing myocardial infarction? The lack of scientific reports on the use of hypoxia in cardiac rehabilitation often results from the fact that most rehabilitation centers do not have access to artificial hypoxia chambers due to the high costs of construction and maintenance. Moreover, specialists often lack sufficient knowledge regarding the safety and potential applications of hypoxia in cardiac rehabilitation.

The main purpose of the research was to analyze the effects of a 22-day training program carried out in normobaric hypoxic conditions corresponding to the altitude of 3000 m a.s.l. in patients after myocardial infarction, and to compare it with the same training conducted in normoxic conditions.

The following research questions were posed in this work:

1. Do the conditions in which rehabilitation training is conducted (normoxia or hypoxia) affect the level of exercise tolerance assessed by an exercise test?
2. Do myocardial hemodynamic indicators assessed by echocardiography change depending on the conditions in which the training program is implemented as part of the second stage of rehabilitation (normoxia or hypoxia)?
3. Does rehabilitation training conducted in different environmental conditions (normoxia or hypoxia) affect the results of blood tests (cytokines, morphology, fibrinogen)?

## 3. Material and Research Methods

### 3.1. Characteristics of the Research Group

This study included 36 patients after myocardial infarction who underwent percutaneous angioplasty with stent implantation. Initially, this study included 38 patients (36 men and 2 women). Due to the low number of female participants, only men were included in the analysis. The characteristics of the respondents are presented in Table 1. They were participants in the second stage of cardiac rehabilitation. Due to the high risk of side effects caused by hypoxic conditions, only patients after uncomplicated myocardial infarction were included in the experiment, who, during the initial endurance test on the treadmill, obtained a result of ≥ 10 MET; the reason for the end was to achieve the target (submaximal, i.e., 85% HRmax) heart rate or fatigue.

The inclusion criteria are as follows:

- a history of uncomplicated myocardial infarction, at least 4 weeks after the incident,
- men and women aged 35–75,
- qualification for cardiac rehabilitation with model A (≥7 MET),
- informed consent to participate in this study,
- no active inflammatory diseases or other uncontrolled non-cardiac diseases.

The exclusion criteria are as follows:

- unstable coronary artery disease,
- recent (up to 4 weeks) past heart attack,
- chronic heart failure,
- arrhythmias and ECG conduction disturbances,
- resistant hypertension,
- positive stress test result,
- atherosclerosis of the peripheral arteries of the lower limbs,
- thromboembolism,
- COPD,
- anemia,
- diseases of the musculoskeletal system that make it impossible to perform an exercise test,
- SARS COVID-19 virus infection,
- lack of consent to participate in this study.

Assignment to training groups, in the condition of normoxia or normobaric hypoxia, was random. Each participant drew a piece of paper marked with the symbol of the group N (normoxia) or H (hypoxia). Participants could withdraw from participation at any time during the experiment.

According to the study protocol, the pharmacological treatment of patients included in this study was optimized and in accordance with the current recommendations for the management of coronary artery disease and myocardial infarction (Table 1). The dosage of drugs was not modified during the experiment. We performed a *t*-test to verify the distribution of medication between the two groups and found no significant difference.

The mean age of the examined patients, the type of myocardial infarction, and the number of implanted stents were similar in both groups (Table 2).

According to the study protocol, the pharmacological treatment of patients included in this study was optimized and in accordance with the current recommendations for the management of coronary artery disease and myocardial infarction. The dosage of drugs was not modified during the experiment.

### 3.2. Methods

The examinations, under normoxic conditions, were performed twice for each patient. The first examination was immediately before the start of the outpatient rehabilitation program (stage II). They were performed on two days: day one, spiroergometric exercise test on a mechanical treadmill, blood collection for laboratory tests; day two, echocardiography of the heart. The same procedure was performed immediately after the end of the 22-day training cycle on days 23 and 24 of the experiment. It was adapted and conducted according to ESC [4] and PCS [5].

The level of exercise tolerance was assessed by a spiroergometric submaximal (85% HRmax calculated from the formula: 220-age) exercise test on a mechanical treadmill. This study used the standard six-step Bruce protocol: Stage 1 = 2.7 km/h, 10%; Stage 2 = 4.0 km/h, 12%; Stage 3 = 5.5 km/h, 14%; Stage 4 = 6.8 km/h, 16%; Stage 5 = 8.0 km/h, 18%; Stage 6 = 8.8 km/h, 20%. The following indicators were analyzed during the test:

- test duration [min],
- distance [m],
- energy cost MET,
- resting (HR_rest_) and peak (HR_peak_) heart rate [bpm],
- resting (SBP_rest_ and DBP_rest_) and peak (SBP_peak_ and DBP_peak_) blood pressure [mmHg],
- peak ventilation VE [1/min],
- BF breathing frequency [1/min],
- peak oxygen consumption VO_2_peak/kg [mL/min/kg],
- respiratory exchange ratio RER.

Throughout the test, until disconnection from the measuring devices, each patient was monitored with a 12-lead ECG set. The examination ended when all analyzed parameters returned to the initial values. Spiroergometric parameters were assessed with the CORTEX METAMAX 3B gas analyzer using the Pulsar treadmill; H/P Cosmos, Nussdorf-Traunstein, Germany. Before tests, the system was turned on at least 20 min earlier, and calibrated before each test according to the manufacturer’s recommendations. Subsequently, gas volume calibration was performed using a standardized 3-L syringe (5530 lot, Hans Rudolph, Inc., Shawnee, MO, USA). Exercise tests ended when the target heart rate (85% HR max) was reached, determined individually for each patient before the test, or due to fatigue. No other reasons (non-physiological) for termination of the study were demonstrated. Due to the safety of patients during the tests, a certified paramedic with a fully equipped professional resuscitation kit and a portable AED was present in the laboratory at all times. During the test day, all patients took their medications as recommended by the physician (cardiologist).

The assessment of heart structures using ultrasonography was carried out by a doctor, a specialist cardiologist trained in echocardiography. ESC echocardiography guidelines were followed for measurements [6]. The GE Vivid Q device was used in this study, and the Simpson 2D method was used to determine the left ventricular ejection fraction. Sector array transducer M5Sc-D. GE software v. 204.

### 3.3. Measured Parameters

The following parameters were measured

- LVEDd—left ventricular end-diastolic dimension (mm)
- LVESd—left ventricular end-systolic dimension (mm)
- LVESV—left ventricular end systolic volume (mL)
- LVEDV—left ventricular end-diastolic volume (mL)
- LVEF—left ventricular ejection fraction (%).

The following tissue Doppler imaging (TDI) indices were also assessed:

- A wave—diastolic muscle movement during atrial contraction [m/s]
- E wave—diastolic movement of the muscle in the phase of rapid filling of the ventricle [m/s]
- e’ lateral—early diastolic velocity of the lateral part of the mitral annulus [m/s]
- e’ septal—early diastolic velocity of the medial part of the mitral annulus [m/s]
- E/E’—ratio of the maximum speed of blood inflow through the mitral orifice in the fast phase ventricular filling and maximum mitral annulus velocity in the phase of quick filling of the chamber
- E/A—ratio of early mitral inflow velocity and mitral inflow velocity over time of atrial contraction
- TAPSE—tricuspid annular plane systolic excursion
- MAPSE—mitral annulus peak systolic excursion.

Laboratory blood tests were performed in a specialized analytical laboratory. In order to determine blood parameters, a nurse collected about 5 mL of venous blood. The material was collected on the first and last day of the experiment. Then, in special containers with ice, it was transported to the laboratory. The following parameters were analyzed: white blood cells (WBC); red blood cells (RBC); hemoglobin (HGB); hematocrit (HCT); platelets (PLT), Sysmex model K400 device; cytokines, TNFα, IL β, IL 10 (Tecan, ELISA kit).

The level of blood oxygen saturation (saturation) during the experiment was assessed at the time of entering the hypoxia chamber, in the 30th minute of adaptation before the start of training, and immediately after the end of training (Pulse Oximeter PMT—T D 8255). All devices used in the research had an EU medical certificate.

### 3.4. Training Protocol

Endurance training in normoxic and hypoxic conditions was carried out in accordance with ESC recommendations every day from Monday to Friday in the mornings. During the experiment, in addition to the patients and two researchers, a paramedic with full resuscitation protection was present in the artificial hypoxia chamber in order to ensure safety. Endurance training in both normoxic and hypoxic conditions was carried out on Kettler ergometers (Kettler Ergo C10). In the conditions of normoxia, the training lasted about 40 min a day. In the case of training under hypoxic conditions, the duration was longer, about 80 min. Before entering the cabin, blood pressure, heart rate, and saturation were measured under normoxic conditions. Then, after entering the chamber, the patients underwent a 30-min adaptation to height (respiratory and circulatory exercises were performed in a sitting position). At the end of the adaptation, the measurements were repeated and training was started. The loads were set individually on the basis of the results of the initial exercise test on the treadmill. Interval training was used with the load increasing every three days, which consisted of the following steps: warm-up, 5 min (minimum initial load 20 W, every three days increase by 5 W); proper training, 35 min (intervals of 4 min work/3 min riding with low load. initially 20 W, increased by 5 W every three days); cooldown, 5 min of riding with a minimum load of 20 W. In this phase, blood pressure, heart rate, and saturation were again measured; rest in a sitting position (stretching exercises, breathing exercises) for about 10 min. The training conditions were as follows:

- normoxia 350 m a.s.l., O_2_ level = 21%, temperature = 21 °C, atmospheric pressure 965 hPa
- hypoxia 3000 m a.s.l., O_2_ level = 14.8%, temperature = 21 °C, CO_2_ level = 1586 ppm, humidity = 33.6%, atmospheric pressure 985 hPa.

Due to a large loss of water in the body during training in the cabin, each patient had a bottle of non-carbonated mineral water.

This study was approved by the Bioethics Committee No. 7/2017 of 18 May 2017. This study was registered in the international Australian New Zealand Clinical Trials Registry, number ACTRN12619001350112, on 1 October 2019.

### 3.5. Statistics

In order to solve the research problem, empirical and exploratory analyses of a comparative and model nature were used. Descriptive statistics (mean values, standard deviations, coefficient of variation, and frequency tables) were used to define and discuss the measurement data matrix. Verification of the occurrence of intragroup differences in individual groups was performed using one-way analysis of variance (ANOVA). When significant statistical differences were present, a post hoc (Tukey’s) test was used.

The presence of between-group differences was determined using multivariate ANOVA with repeated measures. Partial eta-squared (η^2^) was used to represent the effect size for ANOVA tests, where η^2^ indicates: <0.01 small effect, >0.06 medium effect, >0.14 large effect.

To sum up, a complementary analysis of statistical data was carried out using the Statistica computer program (StatSoft Polska Sp. z o.o., Cracow, Poland) and a Microsoft Office Excel spreadsheet (Microsoft, Warsaw, Poland).

## 4. Results

### 4.1. Spiroergometric Exercise Test

Table 3 presents a comparative analysis of the results of an exercise test performed before (I) and after (II) a 22-day training program conducted in normoxic and hypoxic conditions corresponding to an altitude of 3000 m a.s.l.

In each of the analyzed groups of patients, after the completion of the rehabilitation program, exercise tolerance improved. When assessing the range of changes in relation to the environmental conditions prevailing during training, it can be seen that, in the conditions of normoxia, statistically significant improvement was obtained only in terms of energy expenditure MET. Training conducted in hypoxic conditions had a wider impact on changes in the assessed parameters. Significant beneficial changes were demonstrated in relation to test duration, distance covered, energy expenditure MET, respiratory exchange ratio RER, as well as resting values of systolic and diastolic blood pressure.

### 4.2. Morphology, Cytokines, Fibrinogen

Table 4 presents the results of laboratory tests of peripheral blood collected before and after the end of the training programs conducted under normoxic and hypoxic conditions.

In all analyzed groups, after the completion of training, a favorable but statistically non-significant improvement of all results was observed, regardless of the height above sea level at which they were carried out.

### 4.3. Echocardiography with Tissue Doppler

Table 5 presents the results of ultrasound combined with tissue Doppler performed before and after training in normoxic and hypoxic conditions.

Statistical analysis showed favorable changes in the assessed hemodynamic parameters and tissue Doppler in all trainees, regardless of the conditions in which the rehabilitation program was conducted (normoxia or hypoxia). In the case of training conducted under normoxic conditions, statistically significant differences were found only in the end-diastolic dimension (LVEDd). In the group of patients training in conditions corresponding to the altitude of 3000 m a.s.l., significant changes occurred in the diastolic movement of the muscle in the phase of rapid filling of the chamber (E wave).

### 4.4. Determining the Significance of Intergroup Differences Before Training

Table 6 and Table 7 present the results of between-group differences before and after the training programs.

Both before and after training, the significant differences were found only in the case of the IL 10 parameter.

Before the training, the significant differences in eʹ septal and MAPSE parameters were found for the ultrasound examination. After the training, the significant differences were found for LVESd and eʹ septal parameters.

### 4.5. Blood Oxygen Saturation Level

**Figure 1:**
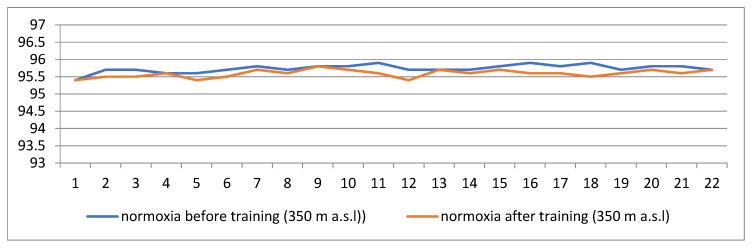
Changes in the value of saturation among the patients training in normoxic conditions corresponding to the altitude of 350 m a.s.l. (immediately before and immediately after exercise).

**Figure 2:**
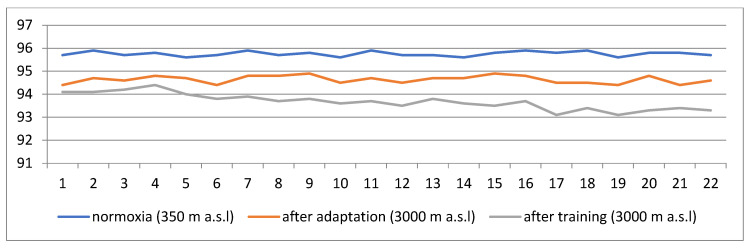
Changes in saturation values among the patients training in hypoxic conditions corresponding to 3000 m a.s.l. (before entering the cabin, after 30 min of adaptation, and after exercise).

Figure 1 and Figure 2 show changes in blood saturation values before entering the hypoxic cabin, after 30 min of adaptation to hypoxic conditions (corresponding to 3000 m a.s.l.), and after the end of training. In the case of training carried out under normoxic conditions, the curves represent the measurements before and after the training.

Figure 1 shows changes in the value of saturation among the patients training in normoxic conditions corresponding to the altitude of 350 m a.s.l. (immediately before and immediately after exercise).

The average range of saturation both before and after the training remained at a constant, correct level of 95.4–95.9%.

Figure 2 shows changes in saturation values among the patients training in hypoxic conditions corresponding to 3000 m a.s.l. (before entering the cabin, after 30 min of adaptation, and after exercise).

The average level of saturation measured before entering the cabin was in the range of 95.6–95.9%. The next measurement made after the 30-min adaptation showed a decrease in blood oxygen saturation to the level of 94.4–94.9%. A clearer decrease in the average saturation level was noted immediately after the end of training. Its range was 93.1–94.4%. The reason, as in the previous case, was the disproportion between the demand for oxygen during exercise and the ability to deliver it at an appropriate level.

## 5. Discussion

### 5.1. Spiroergometric Exercise Test

The effectiveness of cardiac rehabilitation assessed with an exercise test has been repeatedly confirmed in numerous scientific reports [7,8,9]. This was also confirmed in our own research. The group of patients training in normoxic conditions (21% O_2_) improved exercise tolerance in all analyzed test parameters, which undoubtedly proves the effectiveness of the program used, but the level of statistical significance was reached only in relation to the MET metabolic coefficient (*p* = 0.043). In addition to the previously cited authors, a similar effect was also obtained by Piepoli et al. [10], and in patients after myocardial infarction and heart failure by Gianuzzi and Tavazzi [11] and King et al. [12]. Studies confirming the effectiveness of aerobic training in the second stage of rehabilitation, but in the field of alternative training forms, such as indoor cycling [13] hatha yoga [14] or suspension systems [15], have also been presented in the literature. The results obtained by these authors were similar to the results obtained in our own research with a group of patients training in normoxic conditions. As a result of critical stenosis or complete occlusion of the coronary artery, many patients develop a collateral circulation of varying degrees over time, whose task is to supply the peripheral parts of the myocardium. The pace of this development depends on many factors, including the level of earlier (before myocardial infarction) physical activity but, above all, on properly planned and implemented post-infarction rehabilitation. Numerous data indicate that one of the factors that influence the formation of blood circulation is physical exercise, and its effect depends on the dose of exercise. One of the factors influencing the formation of collateral circulation is hypoxia, which, combined with physical exercise, induces angiogenesis and arteriogenesis, which, in turn, cause the formation of new vessels [16]. It has been shown that the presence of a well-developed collateral circulation in the area of the artery responsible for the infarction improves the function of the left ventricle, reduces angina, improves exercise tolerance, and improves the quality of life and distant prognosis, as well as producing a smaller infarct area [17] (The analysis of the results of exercise tests of patients who carried out their rehabilitation program in hypoxic conditions (14.8% O_2_) showed an adequate reaction of their circulation systems to the existing conditions. Despite a significant decrease in oxygen levels in the training room, final exercise tests showed a large increase in exercise tolerance. Compared to the results of patients training in normoxic conditions, the number of assessed parameters for which a statistically significant improvement was found was much greater: longer test duration (*p* = 0.007) and distance covered on the treadmill (*p* = 0.005), increase in MET coefficient (*p* = 0.005, 0.008), decreased RER respiratory exchange rate (*p* = 0.033), and lowered systolic (*p* = 0.035) and diastolic (*p* = 0.001) resting blood pressure values (Table 3). According to some authors, training conducted at high altitudes above the threshold of 2000–3500 m a.s.l. may result in the lack of an increase in maximum heart rate or even its decrease [18] (although Mourot and Millet found, based on clinical studies, that the decrease in HRmax may be smaller in normobaric conditions than in hypobaric hypoxia [19]. Both observations were confirmed in our research because, in the case of training in normobaric conditions as well as in conditions corresponding to the altitude of 3000 m a.s.l., no significant changes in peak heart rate values were obtained. Training in a hypoxic chamber forces the body to adapt to the harsh oxygen conditions in the mountains. Consequently, the circulatory and respiratory systems are provoked to work harder with less oxygen supply. This, in turn, increases the ability to transport oxygen and excrete it through the muscles. Hypoxia also affects the increase in maximum oxygen uptake (VO_2_max) during submaximal and maximum physical effort. This increase was not statistically significant, as in the study by Burtscher et al. [20] According to the authors, intermittent hypoxia increases exercise tolerance by increasing stress resistance and improving oxygen delivery. A three-week period of passive short-term intermittent exposure to hypoxia (14–10% O_2_) resulted in an increase in aerobic capacity and exercise tolerance. The resting heart rate and systolic blood pressure were lowered, which was also demonstrated in our own research (Table 3). Endurance training carried out in a hypoxic environment leads to the dilation of coronary and peripheral blood vessels, reduces arterial stiffness, and improves blood pressure [21] Changes associated with a favorable and, at the same time, statistically significant reduction in the resting values of systolic (*p* = 0.035) and diastolic (*p* = 0.001) blood pressure after the end of training in our own research were observed only in the group of patients training in hypoxic conditions. In this group, as already mentioned, a statistically significant decrease in the RER respiratory exchange rate at the peak of the effort was also observed (Table 3 and Table 6). A score of ≥1.10 is a generally accepted indicator of sufficiently high fatigue (maximum effort) during the test, but exceeding this value is not an indication for its discontinuation. A peak RER < 1.00 reflects submaximal effort and may also be observed with some pulmonary limitations of exercise tolerance [22] However, not all patients with heart failure are able to achieve optimal RER during symptom-limited a CPET (cardio-pulmonary stress test). This is due to skeletal muscle disorders (morphological-structural and functional-metabolic), fatigue of the respiratory muscles leading to improper ventilation, the side effects of drugs, or the occurrence of significant fatigue that makes it impossible to continue the test [23]. In our own research, the correct values of the indicator at the peak of the effort related to a CPET were observed both before and after the applied training program. In the initial study, its value was 1.06, which corresponds to submaximal efforts, while, after the training, it decreased to 0.98, which also proves that the level of submaximal effort has been achieved [22]. This result indicates the better effectiveness of training conducted in conditions corresponding to the altitude of 3000 m a.s.l. for both the cardiovascular and respiratory systems.

Nowadays, altitude training is an increasingly used training method that induces a number of physiological responses. During hypoxia, the body produces hypoxia-inducible factor 1-α (HIF-1-α), causing the formation of new blood vessels, vascularization, and glycolysis, which affect the body’s performance by inducing global tissue hypoxia through training in conditions of lower partial pressure of oxygen (PO_2_) [24,25] Therefore, chronic exposure to moderate altitudes (2000–3000 m) improves the oxygen transport capacity by improving erythropoietin secretion (EPO) and, consequently, increasing the total hemoglobin mass (Mackenzie et al., 2011). This causes an increase in maximum oxygen uptake (VO_2_max) and improved performance. Such adaptation can be observed after 2–3 weeks of exposure to moderate altitudes. Although, we did not measure the EPO concentration in our work, which has been quite well studied under hypoxic conditions. This should be referred to in several works that have shown that the total time spent in hypoxia affects the concentration of EPO. In the one study [26] with continuous exposure to 3000 m (FIO2 15), it was recommended to stay for at least 114 min and, in the case of 4000 m (FIO2 13), for 84 min, which results in an increase in EPO. In the study [27], it was shown that staying for 93.5 min also showed an increase in EPO concentration and an improvement in Vo_2_max after a training intervention. Therefore, it is believed that EPO release is related to two factors FIO2 and duration of volume [25,27]. In our protocol, the patient stayed in the chamber for a total of 55 min during the training session, although taking into account for the density of training units (5 training sessions per microcycle, Mon–Fri), this gives us a total of 275 min of continuous stay during the microcycle, which gives a total of 1100 min of stay in the chamber during 4 microcycles, taking into account the adaptation, warm-up, training, and cool-down.

### 5.2. Blood Parameters

The training carried out in the conditions of normoxia and hypoxia did not significantly affect the obtained results (Table 4). According to the literature, several weeks of exposure to high altitudes and physical effort increase erythropoiesis, produce a greater number of red blood cells, and increase the mass of hemoglobin (Robach et al., 2004) [28]. As a result of acclimatization, the increased level of hemoglobin enables more efficient transport of oxygen to the tissues. This effect is one of the best-known adaptive changes in the body. No differences in the scope of the same assessed blood parameters were found in the studies of Robach et al. [28] and Katayama et al. [29], analyzing the reactions of the body at an altitude of 3000 m a.s.l., as well as Lundby et al. at an altitude of 4100 m a.s.l. [30] Other authors have reported an increase in the concentration of hemoglobin in the blood, which occurred only on the 14th day (of 21 days) in hypoxic conditions [20,31]. An increase in hemoglobin, hematocrit, and red blood cell count has also been noted in other studies two days after the group returned from 1850 m a.s.l. (Suchý et al. [32]. Both conventional and artificially derived specialized training can only significantly increase hemoglobin mass if the following conditions are met: exposure to hypoxia corresponding to an altitude above 2100 m a.s.l. will last more than 14 h a day for at least 3 weeks [33]. In our own research, the lack of significant differences in the morphotic parameters of the blood between the initial and final results was probably caused by too short a time of the daily stay in hypoxic conditions, lasting about 80–90 min with acclimatization, and too long a time interval until the next entry to the cabin (24 h). In the case of hemoglobin and erythrocytes, a slight (non-significant) increase in their level was observed, which could have been a reaction to hypoxia (Table 4).

### 5.3. Cytokines

There are few scientific reports that have assessed the effectiveness of exercises conducted under artificial hypoxia in a group of people with cardiovascular diseases, especially in terms of changes in the level of cytokines. Most studies have focused mainly on healthy and young people or competitive athletes. The nature of the observed changes is completely different than in the case of people after a heart attack. Intensive training conducted at altitudes even above 4000 m a.s.l. causes a large increase in the level of certain cytokines. Therefore, it is difficult to compare the results of our study with similar ones conducted by other authors. Therefore, the analysis was carried out in relation to existing norms in the case of patients after cardiovascular events or to similar thematic works. Richardson et al. conducted a study in a group of 42 untrained people [34]. Intensive interval sprint training was performed in two groups: in normoxic conditions and in hypoxic conditions > 2000 m a.s.l. The third was a control group without training. In the study, the levels of cytokines IL-6 and TNFα were assessed. TNFα increased by 12.9% in normoxic conditions and by 10.8% in hypoxic conditions. In our research, under both normoxic and hypoxic conditions, a slight, statistically non-significant decrease was found (Table 4). Such a reaction could be caused by individually selected training. The loads due to myocardial infarction were much lower and gradually increased during the following days, the duration of the intervals was longer, and each of the participants of the experiment had a 30-min adaptation to the altitude. No changes in the range of, among others, TNFα were found in field studies at an altitude of 2978 m a.s.l. by Edlinger et al. [35] When assessing the level of IL 10 in our research, both hypoxia and normoxia showed a decrease or stabilization of its level. Rohm et al. conducted a study in a group of 12 healthy people, subjecting them to 7 h of exposure to hypoxia in normobaric hypoxic conditions corresponding to the altitude of 5500 m a.s.l. [36] They observed (in the case of IL-10) a statistically significant decrease in the level after hypoxia compared to the baseline value (*p* = 0.016). The amount of pro- and anti-inflammatory cytokines released under the influence of a single effort depends on its intensity and duration as well as the area of active muscles [37]. The greatest changes were observed in response to endurance efforts lasting more than 2 h, i.e., a maximum 60-fold increase in IL-10, and a 3-fold increase in the level of TNFα and IL-1β. Minor changes occurred after submaximal concentric efforts lasting up to 2 h, i.e., a 50% increase in TNFα and no change in IL-1β. Concentric efforts lasting up to 30 min and short-term eccentric efforts induced a much weaker cytokine response, i.e., a maximum 25% decrease in IL-10, a 17% increase in IL-1β, and a lack of change or decrease in the concentration of TNFα [37,38]. This was also partly confirmed in our research in which endurance training was used, carried out both in normoxic and, above all, hypoxic conditions (Table 4). In a sense, this is not only a confirmation of the effectiveness of such training, but a demonstration of the lack of risks associated with a large increase in the level of cytokines in relation to patients after myocardial infarction. After the end of the three-week training program, the levels of the analyzed cytokines changed, but within the normal range. After training in normoxic conditions, no changes in Il-1β and TNFα were found, while in the case of IL-10 a 10% decrease in its level was found. In the case of an altitude of 3000 m a.s.l. the level of IL-10 did not change in relation to the baseline values, while TNFα decreased by about 20%, but without statistical significance (Table 4).

### 5.4. Echocardiography

The dependence of the influence of physical training on the function and structure of the myocardium in patients after myocardial infarction is not fully understood. The prevailing opinion in the literature is that physical training in patients with heart failure does not affect the function of the left ventricle. However, according to some authors, an increase in the efficiency of the heart muscle as a pump under the influence of physical training is possible [39]. The relationship between exercise tolerance and the left ventricular function is complex, because exercise capacity also depends on age, general training, comorbidities, and even mental state [40]. Exercise capacity is improved by training, among other activities, by improving the peripheral perfusion, metabolism, and ultrastructure of skeletal muscles, raising the threshold of anaerobic metabolism [41]. As a result of myocardial infarction or coronary artery disease, the myocardium is impaired and structural changes in the heart muscle occur. Decreased stroke volume (LVESV), end systolic diameter (LVESd), increased end diastolic diameter (LVEDd), or neurohormonal disorders (increased activity of the renin–angiotensin–aldosterone system and catecholamines) may lead to impaired diastolic function of the heart. As a consequence, there is a reduction of the left ventricular ejection fraction (LVEF), which is an indicator of global myocardial contractility and is one of the most important parameters determining the condition of patients after myocardial infarction [42]. In our research, left ventricular indices, significant from the point of view of the effectiveness of the rehabilitation program, were assessed (Table 6 and Table 7). The results show a generally favorable improvement in all echocardiographic parameters, regardless of the conditions in the room (normoxia or hypoxia) during the training sessions. It should be noted that most of these changes took place in the range of the values required for a given parameter. In the case of training in conditions of normoxia, a statistically significant change was noted only in relation to the end-diastolic dimension, which decreased after the training (*p* = 0.00). In a similar study, Sadeghi et al. found no significant changes in LVESd and LVEDd after a longer, 8-week training period in patients with a history of myocardial infarction [43]. The same effect was obtained by Nowak et al. in their study conducted under normoxic conditions during a 6-month observation period [44]. The mean value of the left ventricular ejection fraction assessed in our research underwent a slight increase in both groups (statistically non-significant). Many authors have reported that, among patients with a reduced level of ejection fraction (<50%) as a result of a history of myocardial infarction, and under the influence of the applied improvement programs, this parameter normalized faster than in people with a normal result. Rehabilitation training influences the intensification of angiogenesis and arteriogenesis, which significantly translates into an increase in LVEF and an improvement of its contractility [45]. The mean ejection fraction value before the start of the training program in both assessed groups was normal (>50%). In people whose left ventricular contractility was not significantly impaired as a result of a cardiovascular event with normal EF values, the training caused only small, non-significant changes in this respect. Also, in the case of the end-systolic dimension (LVESd) after the end of both training in normoxic and hypoxic conditions, it was found to have increased, but it was not statistically significant. Analyzing the results of the tissue Doppler, no significant changes were found in the scope of all the assessed indices obtained during the training in the conditions of normoxia (Table 5). Yu et al. used physical training in 127 patients after myocardial infarction with moderate left ventricular diastolic dysfunction for 8 weeks and found a significant increase in E, A, and E/A waves [46]. Skaluba and Litwin analyzed a group of 121 patients with suspected coronary artery disease (CAD) and normal left ventricular ejection fraction who underwent physical training [47]. The authors found that an E/Eʹ ratio > 10 was a strong independent predictor of reduced exercise tolerance. In our research, the average value of this indicator, both before and after training in all three groups exercising in normoxic and hypoxic conditions, was normal (<10). While the conditions of normoxia showed no significant differences in the assessment of tissue Doppler, training in conditions of reduced oxygen content caused significant differences in relation to the E wave (*p* = 0.033). Evaluating the obtained test results, it can be seen that a slightly better effect in the form of improving contractility was obtained after training in hypoxic conditions (Table 5 and Table 7). Such a small improvement was probably due to the very low level of oxygen in the room (14.8%); hence the applied training loads had to be smaller due to concerns about patient safety. This is undoubtedly a valuable clue that already at this stage of the research preliminarily defines the limits of safe training in normobaric hypoxic conditions in patients after myocardial infarction.

### 5.5. Blood Oxygen Saturation Level

The average saturation measurement made before entering the hypoxia cabin was in the range of 95.6–96.2% in both groups. In the case of normoxia, the oxygen content in the room was 21%. Measurements taken daily before and after training showed no differences. The average level of saturation remained at a constant, correct level and ranged from 95.4% to 95.9%. Referring this result to the results of blood tests, it can be seen that the level of erythrocytes and hemoglobin after 22 training sessions did not change compared to the initial values. Due to the reduced oxygen content in the room, training conducted in hypoxic conditions required more effort from patients, although the choice of loads was the same as in normoxic conditions. After 30 min of adaptation to the altitude, in the group of patients training in conditions corresponding to the altitude of 3000 m a.s.l., a significant decrease in the saturation level (94.4–94.9%) was noted and, after the training, a decrease to the level of 93.1–94.4% was noted. The effect of lowering saturation resulted from the disproportion between the demand for oxygen during exercise and the ability to deliver it at the appropriate level for the work performed (Figure 1 and Figure 2).

### 5.6. Study Limitations

Due to the small experimental group, the present research requires continuation. It should be checked whether the visible downward or upward trends of individual parameters will be confirmed in the case of a larger experimental group. Nevertheless, training in conditions of artificial hypoxia, after initial medical qualification, can be successfully used in patients after myocardial infarction.

The described endurance training was temporarily adapted to ESC requirements, which may affect the results. Longer training and exposure to hypoxia would likely produce greater changes. Women should also be included in a future study; their response to hypoxia may be different than in the case of men. In addition, patient recruitment should be conducted only within one center, not several.

Although the results obtained confirm the effectiveness of training, extending the duration of the entire program and the time spent in hypoxia could cause significant changes in the scope of a larger number of analyzed parameters (e.g., echocardiographic).

## 6. Conclusions

1. The conditions in which the rehabilitation training was conducted affect the level of exercise tolerance.
2. The hypoxic conditions in which the training was conducted affected only two hemodynamic parameters: LVESd and e’ septal.
3. Rehabilitation training conducted in various environmental conditions had an impact only on the IL-10 value which, in hypoxic conditions, decreased significantly.

The obtained results highlight the significant role of environmental conditions (hypoxia vs. normoxia) in the adjustment and optimization of cardiac rehabilitation programs, which may contribute to better therapeutic outcomes in patients. This type of training can successfully be used as a complement to the existing rehabilitation plan, enhancing its appeal and increasing its effectiveness.

## Figures and Tables

**Table 1:** Pharmacological treatment of patients in both of groups.

Group of Drugs	Normoxia (*n* = 19)	3000 m n.p.m. (*n* = 17)	*p*
β-blocker	14 (73%)	12 (70%)	0.841
Clopidogrel	5 (26%)	4 (23%)	0.852
Acetylsalicylic acid (ASA)	16 (84%)	14 (82%)	0.885
Statins	14 (73%)	14 (82%)	0.547
α-blocker	1 (5%)	1 (6%)	0.937
Vitamin K antagonists	5 (26%)	3 (18%)	0.545
Angiotensin II receptor antagonists (ARB)	3 (15%)	3 (18%)	0.885
Metformin	3 (15%)	2 (12%)	0.736
Calcium channel blockers	2 (10%)	2 (12%)	0.909
Angiotensin-converting enzyme inhibitors (ACEI)	4 (21%)	4 (23%)	0.355
Diuretics	2 (10%)	1 (6%)	0.626

**Table 2:** Characteristics of the studied groups (assessment of homogeneity).

	Normoxia (*n* = 19)	3000 m a.s.l. (*n* = 17)
Age (years)	58.52 ± 6.09	59.88 ± 5.87
STEMI	8 (43%)	8 (47%)
NSTEMI	11 (57%)	9 (53%)
Number of stents		
1 stent	8 (42%)	8 (43%)
2 stents	5 (26%)	5 (26%)
≥3 stents	6 (31%)	4 (21%)

**Table 3:** Initial and final results of exercise tests in all training groups.

Parameters		Normoxia			3000 m a.s.l.	
Test time [min]	I	9.28 ± 1.39	*F = 1.506* *p = 0.228*	I	9.98 ± 1.11	*F = 8.279* *p = 0.007* *η^2^ = 0.20*
	II	9.56 ± 1.62		II	11.34 ± 1.61	
Distance [m]	I	421.25 ± 113.95	*F = 1.417* *p = 0.242*	I	448.41 ± 107.06	*F = 8.920* *p = 0.005* *η^2^ = 0.22*
	II	443.14 ± 137.39		II	546.66 ± 116.16	
MET [mL/kg/min]	I	8.74 ± 0.66	*F = 4.404* *p = 0.043* *η^2^ = 0.10*	I	8.02 ± 0.87	*F = 7.878* *p = 0.008* *η^2^ = 0.20*
	II	8.96 ± 0.59		II	8.79 ± 0.84	
VE [L/min]	I	88.85 ± 6.53	*F = 0.793* *p = 0.379*	I	87.05 ± 11.62	*F = 2.994* *p = 0.093*
	II	89.81 ± 6.53		II	90.41 ± 9.64	
VO_2peak_ [mL/min/kg]	I	27.17 ± 7.41	*F = 0.263* *p = 0.611*	I	28.45 ± 2.74	*F = 2.218* *p = 0.146*
	II	26.22 ± 5.35		II	29.15 ± 2.61	
BF [L/min]	I	35.24 ± 2.98	*F = 3.934* *p = 0.055*	I	35.68 ± 3.35	*F = 2.216* *p = 0.146*
	II	36.74 ± 2.34		II	36.52 ± 3.35	
RER	I	0.97 ± 0.07	*F = 0.178* *p = 0.675*	I	1.06 ± 0.10	*F = 4.942* *p = 0.033* *η^2^ = 0.13*
	II	0.97 ± 0.07		II	0.98 ± 0.09	
HR_rest_ [L/min]	I	70.60 ± 6.66	*F = 1.320* *p = 0.258*	I	70.41 ± 7.23	*F = 0.000* *p = 1.000*
	II	69.37 ± 7.66		II	70.42 ± 7.46	
HR_peak_ [L/min]	I	132.07 ± 10.96	*F = 0.036* *p = 0.851*	I II	141.91 ± 5.28	*F = 0.143* *p = 0.679*
	II	132.07 ± 10.96			142.29 ± 5.05	
SBP_sp_ [mmHg]	I	126.71 ± 11.92	*F = 0.222* *p = 0.640*	I	133.88 ± 7.43	*F = 4.808* *p = 0.035* *η^2^ = 0.13*
	II	125.41 ± 10.95		II	128.24 ± 7.28	
DBP_sp_ [mmHg]	I	76.71 ± 7.00	*F = 0.329* *p = 0.570*	I	70.52 ± 5.15	*F = 6.297* *p = 0.001* *η^2^ = 0.16*
	II	76.05 ± 7.56		II	66.47 ± 4.93	
SBP_max_ [mmHg]	I	161.05 ± 12.42	*F = 0.017* *p = 0.898*	I	184.26 ± 15.13	*F = 0.381* *p = 0.541*
	II	160.79 ± 13.32		II	185.56 ± 12.11	
DBP_max_ [mmHg]	I	81.31 ± 7.12	*F = 0.000* *p = 1.000*	I	74.71 ± 4.89	*F = 0.258* *p = 0.614*
	II	81.32 ± 7.79		II	75.14 ± 4.99	

**Table 4:** Comparison of the results of blood parameters at the baseline and after the training.

Parameters		Normoxia			3000 m a.s.l.	
WBC	I	6.08 ± 1.15	*F = 0.648* *p = 0.426*	I	7.10 ± 1.12	*F = 0.005* *p = 0.942*
	II	5.93 ± 1.06		II	7.09 ± 1.28	
RBC	I	4.97 ± 0.39	*F = 0.147* *p = 0.703*	I	5.03 ± 0.34	*F = 0.518* *p = 0.476*
	II	4.88 ± 0.32		II	5.09 ± 0.38	
HGB	I	15.11 ± 1.04	*F = 0.085* *p = 0.772*	I	15.41 ± 1.04	*F = 0.660* *p = 0.422*
	II	15.07 ± 1.01		II	15.56 ± 1.03	
HCT	I	45.60 ± 3.25	*F = 0.696* *p = 0.410*	I	45.69 ± 3.23	*F = 1.089* *p = 0.304*
	II	46.04 ± 2.83		II	46.29 ± 2.79	
PLT	I	198.23 ± 47.18	*F = 0.072* *p = 0.790*	I	213.64 ± 38.85	*F = 0.407* *p = 0.527*
	II	200.32 ± 45.31		II	209.64 ± 35.81	
IL 1β	I	48.99 ± 29.91	*F = 0.119* *p = 0.732*	I	30.12 ± 35.19	*F = 0.024* *p = 0.876*
	II	50.69 ± 30.66		II	31.09 ± 35.36	
IL 10	I	3.83 ± 1.08	*F = 1.524* *p = 0.225*	I	4.79 ± 0.02	*F = 0.347* *p = 0.559*
	II	3.62 ± 1.11		II	4.76 ± 0.03	
TNF α	I	10.83 ± 9.79	*F = 0.019* *p = 0.891*	I	11.75 ± 15.06	*F = 0.138* *p = 0.712*
	II	10.61 ± 10.31		II	10.78 ± 11.02	

**Table 5:** Comparison of the results of ultrasound examination with tissue Doppler at the baseline (before) and after training depending on the altitude above sea level at which it was conducted.

Parameters		Normoxia			3000 m a.s.l.	
LVEDd	I	50.05 ± 4.89	*F = 9.560* *p = 0.004* *η^2^ = 0.21*	I	49.32 ± 5.04	*F = 3.783* *p = 0.060*
	II	43.11 ± 8.48		II	47.71 ± 4.73	
LVESd	I	37.68 ± 8.79	*F = 3.972* *p = 0.054*	I	31.61 ± 6.05	*F = 2.544* *p = 0.120*
	II	40.42 ± 9.79		II	33.24 ± 5.40	
LVESV	I	54.65 ± 12.56	*F = 0.047* *p = 0.830*	I	55.88 ± 18.28	*F = 0.039* *p = 0.844*
	II	54.21 ± 10.99		II	55.24 ± 17.20	
LVEDV	I	113.07 ± 18.77	*F = 0.161* *p = 0.690*	I	104.70 ± 29.78	*F = 1.543* *p = 0.223*
	II	114.32 ± 16.36		II	111.00 ± 27.45	
LVEF	I	50.78 ± 5.23	*F = 0.643* *p = 0.428*	I	50.47 ± 6.38	*F = 0.025* *p = 0.874*
	II	51.47 ± 4.66		II	50.65 ± 7.09	
Wave E	I	0.64 ± 0.16	*F = 0.029* *p = 0.865*	I	0.64 ± 0.14	*F = 4.912* *p = 0.033* *η^2^ = 0.13*
	II	0.65 ± 0.18		II	0.77 ± 0.19	
Wave A	I	0.64 ± 0.13	*F = 0302* *p = 0.586*	I	0.68 ± 0.22	*F = 0.293* *p = 0.591*
	II	0.64 ± 0.17		II	0.71 ± 0.21	
e’ lateral	I	0.09 ± 0.02	*F = 0.736* *p = 0.397*	I	0.09 ± 0.03	*F = 0.574* *p = 0.453*
	II	0.09 ± 0.03		II	0.09 ± 0.03	
e’ septal	I	0.06 ± 0.01	*F = 0.795* *p = 0.379*	I	0.09 ± 0.01	*F = 2.433* *p = 0.128*
	II	0.07 ± 0.01		II	0.09 ± 0.02	
E/E’	I	8.27 ± 2.43	*F = 0.553* *p = 0.462*	I	8.18 ± 3.77	*F = 0.435* *p = 0.514*
	II	7.98 ± 2.34		II	8.64 ± 3.46	
E/A	I	1.04 ± 0.39	*F = 0.922* *p = 0.343*	I	1.11 ± 0.48	*F = 0.570* *p = 0.455*
	II	1.10 ± 0.45		II	1.18 ± 0.55	
TAPSE	I	21.81 ± 4.98	*F = 0.125* *p = 0.726*	I	23.61 ± 4.02	*F = 0.960* *p = 0.334*
	II	22.11 ± 5.56		II	22.94 ± 3.86	
MAPSE	I	14.89 ± 2.58	*F = 1.007* *p = 0.322*	I	16.32 ± 3.16	*F = 0.842* *p = 0.365*
	II	15.32 ± 2.38		II	15.82 ± 2.35	

**Table 6:** Spiroergometric treadmill test assessing effect of condition and effect of time.

	Effect of Condition		Effect of Time	
HR_peak_	F = 21.99	*p* = 0.001 *η^2^ = 0.24*	F = 0.12	*p* = 0.729
SBP_peak_	F = 49.91	*p* = 0.001 *η^2^ = 0.22*	F = 0.17	*p* = 0.070
DBP_peak_	F = 7.19	*p* = 0.062	F = 0.09	*p* = 0.778
Time	F = 17.615	*p* = 0.002 *η^2^ = 0.20*	F = 8.591	*p* = 0.004 *η^2^ = 0.11*
Distance	F = 9.336	*p* = 0.003 *η^2^ = 0.14*	F = 8.143	*p* = 0.004 *η^2^ = 0.10*
MET	F = 3.88	*p* = 0.053	F = 3.707	*p* = 0.071
BF	F = 0.363	*p* = 0.546	F = 3.44	*p* = 0.072
RER	F = 4.789	*p* = 0.033 *η^2^ = 0.10*	F = 2.384	*p* = 0.054

*p*-values indicate statistical significance (*p* < 0.05) based on a multivariate repeated-measures ANOVA analysis. Before the training, the significant differences were found in the exercise parameters RER, HR_peak_, SBP_rest_, and DBP_peak_. After the training, the significant differences were obtained in terms of test time, DBP_rest_, and SBP_peak_.

**Table 7:** Results of echocardiography between-group assessing effect of condition and effect of time before and after the training programs.

	Effect of Condition		Effect of Time	
Parameter				
e-septal	F = 66.138	*p* > 0.000 *η^2^ = 0.49*	F = 3.357	*p* = 0.07
LVEDD	F = 3.707	*p* = 0.059	F = 12.75	*p* = 0.004 *η^2^ = 0.16*
LVESD	F = 12.142	*p* > 0.000 *η^2^ = 0.15*	F = 6.256	*p* = 0.014 *η^2^ = 0.08*
MAPSE	F = 4.418	*p* = 0.040 *η^2^ = 0.06*	F = 0.013	*p* = 0.967 *η^2^ = 0.00*

*p*-values indicate statistical significance (*p* < 0.05) based on a multivariate repeated-measures ANOVA analysis.

## Data Availability

The datasets generated and analyzed during the current study are not publicly available due to decision of all coauthors but are available from the corresponding author on reasonable request.
